# Analysis of eight genes modulating interferon gamma and human genetic susceptibility to tuberculosis: a case-control association study

**DOI:** 10.1186/1471-2334-10-154

**Published:** 2010-06-07

**Authors:** Marlo Möller, Almut Nebel, Paul D van Helden, Stefan Schreiber, Eileen G Hoal

**Affiliations:** 1 Molecular Biology and Human Genetics, MRC Centre for Molecular and Cellular Biology and the DST/NRF Centre of Excellence for Biomedical TB Research, Faculty of Health Sciences, PO Box 19063, Stellenbosch University, Tygerberg 7505, South Africa; 2 Institute for Clinical Molecular Biology, Christian-Albrechts-University, Schittenhelmstrasse 12, 24105 Kiel, Germany

## Abstract

**Background:**

Interferon gamma is a major macrophage-activating cytokine during infection with *Mycobacterium tuberculosis*, the causative pathogen of tuberculosis, and its role has been well established in animal models and in humans. This cytokine is produced by activated T helper 1 cells, which can best deal with intracellular pathogens such as *M. tuberculosis*. Based on the hypothesis that genes which regulate interferon gamma may influence tuberculosis susceptibility, we investigated polymorphisms in eight candidate genes.

**Methods:**

Fifty-four polymorphisms in eight candidate genes were genotyped in over 800 tuberculosis cases and healthy controls in a population-based case-control association study in a South African population. Genotyping methods used included the SNPlex Genotyping System™, capillary electrophoresis of fluorescently labelled PCR products, TaqMan^® ^SNP genotyping assays or the amplification mutation refraction system. Single polymorphisms as well as haplotypes of the variants were tested for association with TB using statistical analyses.

**Results:**

A haplotype in interleukin 12B was nominally associated with tuberculosis (p = 0.02), but after permutation testing, done to assess the significance for the entire analysis, this was not globally significant. In addition a novel allele was found for the interleukin 12B D5S2941 microsatellite.

**Conclusions:**

This study highlights the importance of using larger sample sizes when attempting validation of previously reported genetic associations. Initial studies may be false positives or may propose a stronger genetic effect than subsequently found to be the case.

## Background

Infection with the tuberculosis (TB) agent *Mycobacterium tuberculosis *(*M. tuberculosis*) and its subsequent outcomes (active TB, latent infection or clearance of the bacterium by the pulmonary immune system) are complex traits due to interactions between numerous host genetic susceptibility factors and the environment. Heritability analyses have shown that an individual's immune response to TB infection will be regulated by the genetic background, with an estimated heritability ranging from 36% to 80% [1-6]. Although many of the host genetic factors involved in TB still remain unidentified, several susceptibility genes have been repeatedly associated with the disease in different populations [7,8].

Interferon gamma (IFN-γ) is a member of the interferon family which plays a crucial role in the reaction of the immune system in resistance to pathogens such as *M. tuberculosis*. There has been a great deal of interest in this cytokine since its discovery, because the macrophage, an important target cell of IFN-γ, is a fundamental role player in the immune system. The T helper 1 (Th1) cell response, which is required to contain *M. tuberculosis *infection, is largely characterised by IFN-γ production. However production of this cytokine alone is not sufficient to protect against disease [9]. Even so, convincing evidence for its importance in the control of mycobacterial infections has been found in both experimental and clinical studies. Mice with a disrupted IFN-γ gene (*IFNG*) show increased susceptibility to TB [10] and replacement of the gene into the lung confers resistance [11]. In most TB patients the production of *M. tuberculosis*-induced IFN-γ by peripheral blood mononuclear cells is reduced at the time of diagnosis [12]. During and after successful treatment, these levels increase significantly [13]. It is also known that the IFN-γ concentrations in sputum and bronchoalveolar lavage fluid can be used as an estimate of disease activity via a direct correlation [14]. Although IFN-γ is required for an initial protective Th1 cell response to *M. tuberculosis*, the increased production of this cytokine post-infection is indicative of the risk of developing active TB. A recent study showed that macaque monkeys with high IFN-γ levels two months post infection with *M. tuberculosis *were more likely to develop active TB [15] and similar observations have been made in humans [16,17], perhaps indicating a failed immune response. Some experimental data have suggested that IFN-γ is a correlate of protective immunity against TB [18,19], although other studies in humans, mice and cattle do not support this [20-22]. Humans with mutations in genes of the interleukin 12/interleukin 23/IFN-γ axis have an increased susceptibility to even non-pathogenic mycobacteria and are extremely susceptible to *M. tuberculosis *and *Salmonella*, but not to other bacteria (reviewed by [23]). These mutations are all associated with the rare human syndrome known as Mendelian susceptibility to mycobacterial disease (MSMD).

In addition to the experimental and clinical studies discussed above, genetic association studies have also suggested that IFN-γ is important in protecting against mycobacterial infection. The functional +874A→T single nucleotide polymorphism (SNP), a common variant in the *IFNG *gene, has been associated with TB in the South African Coloured population (SAC) [24] and various others [25] and even appears to be clinically relevant in sputum conversion in patients [26]. However, since TB is a complex disease and several genes are involved in its pathogenesis, it is possible that genes regulating IFN-γ levels could also contain variants contributing to susceptibility.

Given the clinical and experimental evidence showing a crucial role of the IFN-γ pathway in host defence against TB, we investigated eight candidate genes which could potentially modulate the function of this vital cytokine, namely interleukin 4 (*IL4*), interleukin 10 (*IL10*), interleukin 12B (*IL12B*), interleukin 12 receptor beta 1 (*IL12RB1*), interleukin 12 receptor beta 2 (*IL12RB2*), interleukin 18 (*IL18*), wingless-type MMTV integration site family, member 5A (*WNT5A*) and frizzled homolog 5 (*FZD5*). Fifty-four polymorphisms in these genes were genotyped and evaluated using a case-control association analysis in the SAC population. We found a novel allele of the *IL12B *microsatellite D5S2941 and showed the significance of using larger sample sizes when attempting validation of previously reported genetic associations.

## Methods

### Study population

This study was carried out in the Cape Town metropolitan area of the Western Cape Province of South Africa, where TB is endemic and the estimated prevalence of the human immunodeficiency virus (HIV) among adults is approximately 2% [27]. The incidence of TB in this province was 1005 per 100 000 population during 2007 [28]. All study participants were from the SAC population. This unique population is a group of mixed ancestry, which dates back several generations, and has San, Khoi, Malaysian, African black and European genetic contributions. In a previous study no evidence of significant population stratification was found for this population (p = 0.26) [29]. Informed consent was obtained from all subjects included in this study. Blood samples were collected with the approval of the Ethics Committee of the Faculty of Health Sciences, Stellenbosch University (project number 95/072), and DNA purified by standard methods. Known HIV positive individuals were excluded from the study. Patients (n = 432, age in years = 34 ± 14.8, males = 53%) were confirmed as having TB by bacteriological analyses. Controls (n = 482, age in years = 27 ± 12.3, males = 23%) were healthy individuals with no history of TB who live in the same high incidence community as the patients and were therefore most likely exposed to the bacterium.

### Genotyping

Polymorphisms were selected from the literature (based on previous associations (Additional file 1) or functional effects) and dbSNP (Additional file 2). Genotyping of the 54 variants was done by the SNPlex Genotyping System™ (Applied Biosystems), capillary electrophoresis of fluorescently labelled PCR products [30], TaqMan^® ^SNP genotyping assays (Applied Biosystems) [31,32] or the amplification mutation refraction system (ARMS-PCR) [33].

### Statistical analysis

Since the allele frequencies of the polymorphisms analysed in this study were not known for the SAC population prior to the completion of genotyping and were necessary for power calculations before starting the experiment, we estimated from prior data that each SNP would at least have a minor allele frequency of 5% in our study group. Given this assumption we had 95% confidence (alpha error p = 0.05) and 80% power (beta error = 0.2) to detect an odds ratio of at least 2.15 with the number of samples available (432 cases and 482 controls). After genotyping, power calculations were done with the experimentally determined allele frequencies of the SNPs previously associated with tuberculosis to confirm that we had enough power to exclude the previously reported genetic effect sizes in the SAC population. All power calculations were done with Epi Info 2000 (Centers for disease control and prevention, USA). Hardy-Weinberg equilibrium was assessed for all SNPs.

Contingency tables of the distribution of genotypes between cases and controls were analysed by the chi-square test or the Fisher's exact test where appropriate. For the *IL12B *D5S2941 (rs10631390) microsatellite marker the number of ATT repeats was determined by direct counting and plotted as a distribution graph. Since this graph was bimodal, the alleles were divided into two subclasses, as previously described [34-36]. The shorter repeats, with (ATT)_7 _and (ATT)_8_, were designated as S alleles and the longer repeats, with (ATT)_9 _and (ATT)_10_, were designated as L alleles. Prism version 4.02 was used to calculate p-values for single-point associations (Additional file 2).

Bonferroni corrections for multiple testing were done by considering the number of independent linkage disequilibrium (LD) blocks per gene [37]. We determined twenty-one independent tests and consequently a p-value of 0.002 was adopted as a threshold for significance. Haplotype frequencies were inferred using the COCAPHASE program that is part of the UNPHASED suite [38], http://portal.litbio.org/Registered/Webapp/glue/). Haplotype blocks were selected with Haploview [39] by considering LD blocks. Global significance for haplotype analyses were tested with COCAPHASE and 10 000 permutation replications were done.

## Results and discussion

We genotyped 53 SNPs and 1 short tandem repeat (STR) in a large collection of SAC TB patients (n = 432) and controls (n = 482). All variants were in Hardy-Weinberg equilibrium in the control population. Four SNPs (rs2070874 in *IL4*, rs2853639 in *IL12B *and rs566926 and rs7622120 in *WNT5A*) were nominally associated with disease (p < 0.05), but these were no longer considered to be statistically significant after adjusting our threshold of significance (p = 0.002) to correct for multiple tests (Additional file 2). More importantly, however, our study did not replicate previously reported associations of *IL4*, *IL10*, *IL12B *and *IL12RB1 *polymorphisms with TB (Additional files 1 and 2).

Polymorphisms in the *IL4 *promoter could influence the transcription levels of that gene. Specifically, a functional SNP (rs2243250, IL-4-C590T), located 589 bp upstream of the transcriptional site, has been associated with increased promoter strength, stronger binding of transcription factors and with different levels of IL-4 activity [40,41], but was not associated with TB in our study. The CC genotype of this polymorphism was previously associated with protection against pulmonary TB in south India and Russia [42,43], but not in The Gambia [44]. A possibility is that alternative splicing, and not increased expression of the *IL4 *gene, is the actual regulatory mechanism in the production of IL-4, since the product of alternative splicing, namely IL-4δ2, is a competitive antagonist of IL-4 [45]. Individuals with latent TB infection, but who remain healthy, have high levels of this variant mRNA [46]. During chemotherapy, IL-4δ2, but not IL-4, levels increased in HIV positive and negative patients with TB [47]. In addition, there is a difference in the stability of the two *IL4 *mRNA products in TB patients, with IL-4 being more stable than IL-4δ2 [48]. Therefore the ratio of IL-4 and IL-4δ2 may play a role in disease progression, treatment or outcome, rather than the phenotype "diseased" per se [48]. An example of a gene involved in response to TB treatment is the vitamin D receptor gene (*VDR*). The time taken for an individual to convert to sputum negativity while receiving TB chemotherapy could be independently predicted by the *VDR *genotype, even though the *VDR *polymorphisms were not associated with TB disease [49]. The apparent exclusion of a significant genetic effect of *IL4 *in TB susceptibility is therefore not an indication that the cytokine does not have a function in TB disease.

The three most frequently investigated polymorphic variants in *IL10 *(rs1800872, rs1800871 and rs1800896) were not associated with TB in our study. These SNPs are linked and in most cases three haplotypes (CCG, ATA and CCA) are observed [50]. The haplotypes and individual SNPs have been shown to correlate with IL-10 production, transcriptional activity and nuclear-binding activity [50-53]. The rs1800896 SNP has previously been associated with TB in Cambodia [54], Sicily [55] and Turkey [56], but not in China [57], The Gambia [58], Malawi [59], India [60], Spain [61] or Korea [62]. A smaller, second study done in Korea found that this polymorphism was associated with new and recurrent TB [63]. A recent meta-analysis found no evidence for association between TB and this SNP [25]. The rs1800872 SNP is in complete linkage disequilibrium (LD) with rs1800871 and these SNPs were associated with TB in Korea [62], but not in The Gambia, Malawi, China, Colombia, Turkey or Uganda [56-59,64-66]. Since the three-SNP haplotype of *IL10 *was previously associated with TB, we also tested this in the SAC population. In contrast to other studies [56,65], there was no association evident in our analysis (Table 1). A four-SNP haplotype previously associated with TB in Korea [62], consisting of rs1800896, rs1800871, rs1800872 and rs3024496, was also not associated with disease in this study (Table 2). Stein *et al. *found that a three-SNP haplotype consisting of rs1518111, rs1554286 and rs1800872 was associated with protection against TB [64]. More recently a large case-control association study (number of cases = 2010, number of controls = 2346) in Ghana determined that an *IL10 *haplotype was in fact associated with tuberculin skin test (TST) response and not with pulmonary disease [67]. This could not be tested for in our study.

**Table 1:** *IL10 *three SNP haplotype analysis.

Block 1^a^		Frequency Cases	Frequency Controls	p value
1	C-C-A	0.37	0.34	0.10

2	A-T-A	0.31	0.34	0.08

3	C-C-G	0.32	0.32	0.92

**Global significance^b^**				0.15

^a ^The order of the SNPs is rs1800872, rs1800871 and rs1800896.

^b ^Global significance were calculated in COCAPHASE [38] and 10 000 permutations were done.

**Table 2:** *IL10 *four SNP haplotype analysis.

Block 1^a^		Frequency Cases	Frequency Controls	p value
1	T-A-T-A	0.31	0.34	0.12

2	C-C-C-G	0.29	0.29	0.91

3	C-C-C-A	0.20	0.18	0.18

4	T-C-C-A	0.17	0.16	0.36

5	T-C-C-G	0.03	0.03	0.81

**Global significance^b^**				0.44

^a ^The order of the SNPs is rs3024496, rs1800872, rs1800871, rs1800896.

^b ^Global significance were calculated in COCAPHASE [38] and 10 000 permutations were done.

**Figure 1:**
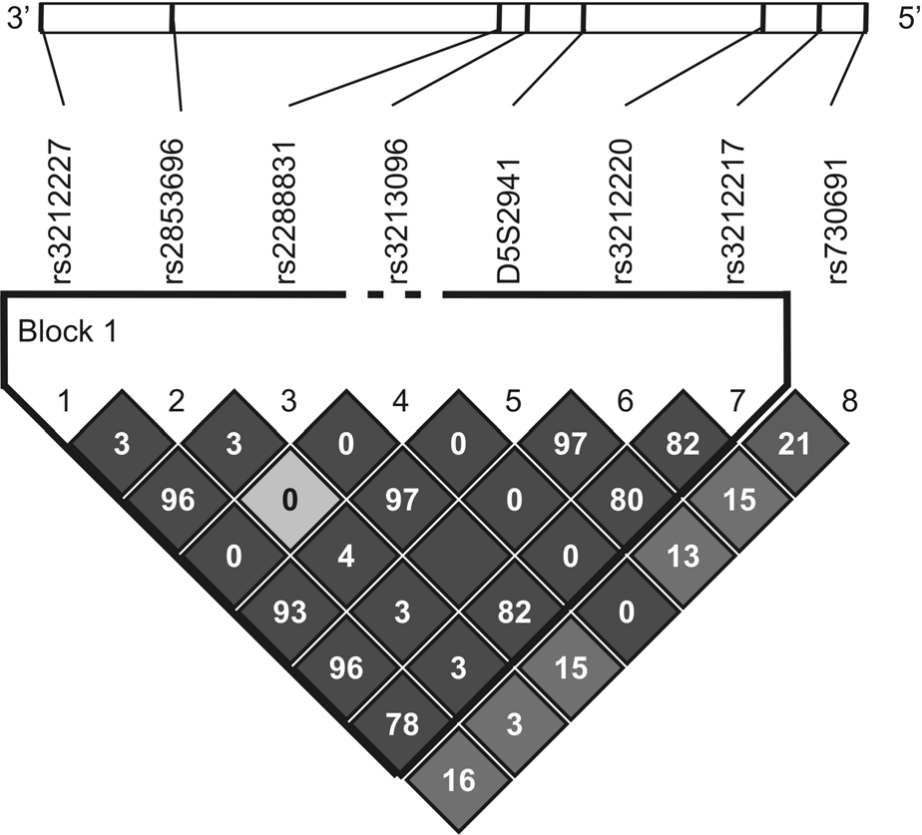
**Plot of LD between *IL12B *markers analysed in control individuals of the SAC population**. Generated by Haploview v4.1. The 5' and 3' ends of the genes are indicated and r^2 ^values (%) are shown on the squares (no value = 100%). The colours of the squares represent D' values, with dark grey being D' = 1, and white D' = 0.

The *IL12B *D5S2941 microsatellite (rs10631390), an (ATT)_n _repeat in intron 2 of the gene, was previously associated with TB in the Hong Kong Chinese population [68]. We identified a novel (ATT)_10 _allele (which was confirmed by sequencing, data not shown) as well as the known (ATT)_7_, (ATT)_8 _and (ATT)_9 _alleles of the D5S2941 microsatellite in the SAC population. However, none of these were associated with TB in our study (Additional file 2). In previous studies done in Caucasian-Americans [69] and Hong Kong Chinese [68], only the (ATT)_8 _and (ATT)_9 _alleles of this STR were ever observed, although the presence of the (ATT)_7 _allele in two Swedish families was mentioned in a diabetes study [30]. There are no reports concerning this marker in African populations. Since the (ATT)_7_, (ATT)_8 _and (ATT)_9 _alleles were previously identified in individuals from European and Asian descent, we speculate that the (ATT)_10 _allele is a genetic contribution from the African parental population of the SAC. The 3'UTR *IL12B *SNP (rs3212227) [70,71] may influence gene expression levels and has previously been associated with TB in various populations [68,72-74], but not in all [60,75,76]. This polymorphism was not associated with TB in our study either. Even though none of the other *IL12B *polymorphisms investigated were associated with TB in the single-point analysis (similar to the results published by Kusuhara *et al. *[77] for rs11135058, rs2288831 and rs6870828), we found a nominally significant association between a haplotype in *IL12B *and resistance to TB in the SAC population (Figure 1, Table 3). The haplotype occurred more frequently in controls than in cases (p = 0.02, OR = 1.53, 95%CI [1.07-2.02]) and was tagged by the A allele of the rs2853696 SNP, which was not associated with TB on its own. However, after permutation testing this association was not globally significant (p = 0.11).

**Table 3:** Haplotype analysis of *IL12B*.

Block 1^a^		Frequency Cases	Frequency Controls	p value
1	A-G-T-S-G-G	0.63	0.62	0.61

2	C-G-C-L-T-C	0.25	0.24	0.49

**3**	**A-A-T-S-G-G**	**0.06**	**0.09**	**0.02**

4	C-G-C-L-T-G	0.03	0.04	0.38

**Global significance^b^**				0.11

^a ^The order of the SNPs in each block corresponds to Figure 1, with D5S2941 included and rs3213096 excluded due to its low allele frequency in the SAC population.

^b ^Global significance were calculated in COCAPHASE [38] and 10 000 permutations were done.

Akahoshi *et al. *[78] reported the first case-control association study assessing *IL12RB1 *in TB susceptibility in the general population. Two common haplotypes (consisting of 4 SNPs, of which rs375947 was genotyped in our study) were identified, and homozygosity for the allele 2 haplotype was significantly associated with TB in Japan. Healthy subjects with this haplotype had lower levels of IL-12-induced signalling. Remus *et al. *[79] investigated *IL12RB1 *in 101 Moroccan families where two promoter polymorphisms in strong LD with each other (rs436857 and rs393548) were associated with disease, but no association was detected with the haplotype reported by Akahoshi *et al. *[78]. A second study done in Japan showed that two intronic SNPs were associated with disease, and a haplotype consisting of different SNPs to that identified by Akahoshi *et al.*, was associated with resistance to TB [77]. However, this study did not replicate the association found with the promoter polymorphisms in the Moroccan families. Our investigation of the SAC population, genotyping larger sample sizes than the two positive reports from Japan [77,78], did not detect any association with *IL12RB1 *SNPs (Additional file 2) or haplotypes (data not shown). Studies in Korea [80] and Indonesia [81] could not validate the findings either. The association found in Japan could be population-specific, but it could also a false-positive result. For this reason, replication of those results should be attempted in an independent, larger Asian population [81].

Promoter polymorphisms in *IL12RB2 *were also investigated, since this gene was previously associated with leprosy [82], an infectious disease caused by *Mycobacterium leprae*. Coding SNPs in this gene were previously shown to have no influence on mycobacterial infection [78,80], but the degree of expression of this gene, possibly regulated by promoter polymorphisms, could determine the intensity of the cell-mediated immune response to mycobacteria [82]. However rs3762317, which disrupts a GATA transcription factor binding site [33], and rs11576006, which participates in the creation of another GATA site [33], were not associated with TB in our study.

IL-18 is a proinflammatory cytokine and, together with IL-12, one of the primary inducers of IFN-γ production by T cells [83,84]. To date only one other association study between polymorphisms in *IL18 *and susceptibility to TB has been published, namely a study by Kusuhara *et al. *which considered 21 candidate genes including *IL18 *[77]. Amongst others, they genotyped 6 SNPs spread throughout *IL18*, but did not consider the functional promoter or synonymous polymorphisms, and found no association between this gene and TB susceptibility. We genotyped functional promoter polymorphisms, the synonymous SNP and other variants, but did not detect statistically significant associations in single SNP or haplotype analyses either.

Both the innate and acquired immune systems are necessary to eradicate mycobacteria from the host [20]. A microarray-based gene-expression screening of mycobacteria-infected macrophages was done to search for novel regulatory pathways in innate responses to infection [85]. This study suggested that the Wingless/Frizzled (WNT/FZD) signalling system connects the innate and adaptive immunity during infections and implicated the WNT5A protein in human defence against infection with *M. tuberculosis *[85]. Blumenthal *et al. *[85] demonstrated that WNT5A is expressed by antigen-presenting cells when they are stimulated by mycobacteria or other bacterial structures. In addition, both WNT5A and its receptor FZD5, regulate IL-12 and IFN-γ production in antigen-presenting cells when exposed to mycobacteria. None of the *WNT5A *or *FZD5 *SNPs genotyped was associated with disease in the case-control. In addition, no haplotypes from these genes were associated with TB either.

Perhaps surprisingly, our study did not validate some previously described findings, even though it had enough power to detect the effect sizes reported by those studies (Additional file 2). Some of those associations were based on extremely low sample numbers (which could lead to false positive associations) or did not correct for multiple testing. Underpowered studies may not detect effect sizes that are small, but reasonable considering the current understanding of the host genetics of complex diseases [86]. The question of correcting for multiple tests (and which method to use) is a contentious one [87] and it is often bypassed. However, the lack of reproducibility of certain associations could also be a result of ethnic-specific associations. Alternatively, polymorphisms could have smaller effect sizes in the SAC population than we were able to detect with our sample (see Statistical Analysis and Additional file 2). It is also probable that the polymorphisms studied here are associated with primary infection in the SAC population, a hypothesis which we would not be able to test due to the high incidence of latent TB infection in the control community where sample collection was done. Our study was more likely to test the possible associations of the SNPs with TB progression from latent infection to active disease only, but we cannot rule out the possibility that some controls were not TST positive as TSTs were not done. However, our previous study of healthy children and young adults from the control community found that 80% of children older than 15 years had positive tuberculin skin tests, an indication of latent infection with *M. tuberculosis *[88].

The findings presented here demonstrate the common phenomenon in association studies, where the first report is usually a positive association and subsequent studies are often negative. Unfortunately, because of publication bias, other association studies which considered these eight candidate genes and found results similar to ours may not have been published.

## Conclusions

We found a nominally significant association with an *IL12B *haplotype which was not considered to be globally significant after permutation testing to determine the significance for the entire analysis, and we identified a novel allele of the *IL12B *D5S2941 microsatellite in the SAC population. This research illustrates the complexity of TB where a well-known pathway cannot be conclusively genetically associated with the disease.

## Competing interests

The authors declare that they have no competing interests.

## Authors' contributions

The work presented in the article was carried out in collaboration between all authors. MM participated in the study design and genotyping experiments, analysed the data, interpreted the results and wrote the paper. AN, PDVH, SS and EGH participated in the study design, interpreted results and wrote the paper. All authors approved the final manuscript.

## Pre-publication history

The pre-publication history for this paper can be accessed here:

http://www.biomedcentral.com/1471-2334/10/154/prepub

## Supplementary Material

Additional file 1 **Previous association studies of tuberculosis susceptibility candidate genes investigated in this study**. A table which summarises previous association studies of candidate genes investigated in this study. Click here for file

Additional file 2 **Candidate genes and polymorphisms analysed in this study**. A table containing the results of the single-point association analyses done in this study. Click here for file
