# Supplementary material for: Analysis of eight genes modulating interferon gamma and human genetic susceptibility to tuberculosis: a case-control association study
Source: BMC Infect Dis. 2010 Jun 7;10:154. doi: 10.1186/1471-2334-10-154 (PMC2891757; doi:10.1186/1471-2334-10-154)
Supplement: Additional file 1 — Previous association studies of tuberculosis susceptibility candidate genes investigated in this study. A table which summarises previous association studies of candidate genes investigated in this study. [file 1471-2334-10-154-S1.PDF]

Additional file 1 - Previous association studies of tuberculosis susceptibility candidate genes investigated in this study.

Gene	Polymorphism	Allele	Population	Phenotype	Cases	Controls	p value (p_c)[a]	OR[b]	Reference
IL4	rs2243250 (-590C/T)	CT	India (south)	pTB[c]	129	127	**< 0.01**		[1]
			Gambia	TB	210	220	ns		[2]
		CC,CT	Russia (Tomsk)	pTB	78	82	**0.008, 0.003**	**2.14, 1.77**	[3]
IL10	H[d]: rs1800896-rs1800871-rs1800872	ACC/ATA	Turkey	TB[e]	81	50	0.02 (0.11)		[4]
		GCC/ACC	Turkey	TB	81	50	0.04 (0.22)		[4]
		GCC	Turkey	TB	128	80	**0.000 (0.000)**	**2.22**	[5]
		ACC	Turkey	TB	128	80	**0.002 (0.004)**	**0.53**	[5]

Gene	Polymorphism	Allele	Population	Phenotype	Cases	Controls	p value (p_c)[a]	OR[b]	Reference
	H: rs1800896-rs1800871-rs1800872- rs3024496	ACCT	Korea	TB	459	871	**0.04**	**0.75**	[6]
	H: rs6703630-rs1800896-rs1800871-rs1800872	AACC	Ghana	TST[f] response	2010	2346		**2.15**	[7]
	H: rs1518111- rs1554286-rs1800872	TAA	Uganda	TB	333 families		**0.002**		[8]
	Microsatellite		Gambia	pTB	401	408	ns[g]		[9]
	rs1800871		Malawi	TB	210	698	ns		[10]
			Colombia	pTB	140	135	ns		[11]
			Turkey	TB	81	50	ns		[4]
			Turkey	TB	128	80	ns		[5]
	rs1800871/ rs1800872		Gambia	pTB	401	408	ns		[9]
			China (Hong	TB	385	451	ns		[12]

Gene	Polymorphism	Allele	Population	Phenotype	Cases	Controls	p value (p_c)[a]	OR[b]	Reference
			Kong)						
	rs1800872		Malawi	TB	210	705	ns		[10]
		C	Korea	TB	459	871	**0.005**	**0.69**	[6]
			Colombia	pTB	140	135	ns		[11]
			Turkey	TB	81	50	ns		[4]
			Uganda	TB	333 families		ns		[8]
			Turkey	TB	128	80	ns		[5]
	rs1800896	G	Gambia	pTB	401	408	ns		[9]
		AG	Cambodia	pTB	358	106	**0.01**	**1.84**	[13]
			Spain	pTB	113	207	ns		[14]
		A	Sicily	pTB	45	100	**Significant**		[15]
		GG	Malawi	HIV+ [h] TB	155	541	**0.007**	**0.37**	[10]
			China (Hong	TB	385	451	ns		[12]

**Gene**	**Polymorphism**	**Allele**	**Population**	**Phenotype**	**Cases**	**Controls**	**p value (p_c)[a]**	**OR[b]**	**Reference**
			Kong)						
			Korea	TB	459	871	ns		[6]
		G	Turkey	TB	81	50	**0.014**	**2.02**	[4]
		A	Colombia	pTB	140	135	**0.02**		[11]
		AA	Korea	New TB	80	117	**<0.001**	**20.7**	[16]
		AA	Korea	Recurrent TB	65	117	**<0.01**	**6.9**	[16]
			India (south)	pTB	132	143	ns		[17]
		G	Turkey	TB	128	80	**0.000 (0.000)**	**2.22**	[5]
			Meta-analysis	TB	8 studies		ns		[18]
	rs3024496		Korea	TB	459	871	ns		[6]
IL12B	D5S2941	$(ATT)_8$	China (Hong	TB	516	514	**<0.001**	**2.14**	[19]

Gene	Polymorphism	Allele	Population	Phenotype	Cases	Controls	p value (p_c)[a]	OR[b]	Reference
			Kong)						
	H: Promoter-rs3212227	22-11	India	TB	123	89	**0.0009**		[20]
	rs11135058		Japan	TB	87	265	ns		[21]
	rs2288831		Japan	TB	87	265	ns		[21]
	rs3212227		Russia	pTB	58	127	ns		[22]
			Caucasian US	pTB	106	107	ns		[23]
			African American	pTB	180	167	ns		[23]
		AA	China (Hong Kong)	TB	516	514	**0.027**	**1.79**	[19]
			Japan	pTB	114	110	0.03 (ns)	0.50	[24]
		C	Russia	TB	304	129	**0.044**		[25]
			India (south)	pTB	132	143	ns		[17]

Gene	Polymorphism	Allele	Population	Phenotype	Cases	Controls	p value (p_c)[a]	OR[b]	Reference
	rs6870828		Japan	TB	87	265	ns		[21]
IL12RB1	H: rs11575934-rs17852635-rs375947-rs401502	GTCC	Japan	TB	98	197	**0.013**	**2.45**	[26]
	1781G>A		Indonesia	TB	382	437	ns		[27]
	rs11575926		Indonesia	TB	382	437	ns		[27]
	rs11575932		Korea	TB	115	151	ns		[28]
	rs11575934		Japan	pTB	114	110	ns		[24]
			Korea	TB	115	151	ns		[28]
			Indonesia	TB	382	437	ns		[27]
		GG	Japan	TB	87	265	**0.0078**	**2.53**	[21]
	rs11575935		Korea	TB	115	151	ns		[28]
			Indonesia	TB	382	437	ns		[27]
	rs375947		Korea	TB	115	151	ns		[28]

Gene	Polymorphism	Allele	Population	Phenotype	Cases	Controls	p value (p_c)[a]	OR[b]	Reference
		CC	Japan	TB	87	265	**0.0032**	**2.83**	[21]
	rs393548	AA	Morocco	TB	101 families		**0.019**	**2.03**	[29]
	rs401502		Korea	TB	115	151	ns		[28]
			Indonesia	TB	382	437	ns		[27]
		CC	Japan	TB	87	265	**0.0032**	**2.83**	[21]
	rs436857	CC	Morocco	TB	101 families		**0.013**	**2.69**	[29]
			Indonesia	TB	382	437	ns		[27]
IL12RB2	rs12142823		Japan	pTB	114	110	ns		[24]
	rs1546159		Japan	TB	87	265	ns		[21]
	rs2252596		Japan	TB	87	265	ns		[21]
	rs6685568		Japan	TB	87	265	ns		[21]
	rs7518845		Japan	TB	87	265	ns		[21]
	rs7535591		Japan	TB	87	265	ns		[21]

Gene	Polymorphism	Allele	Population	Phenotype	Cases	Controls	p value (p_c)[a]	OR[b]	Reference
IL18	rs1946518		India (south)	pTB	165	173	ns		[30]
	rs187238		India (south)	pTB	165	173	ns		[30]
	rs3882891		Japan	TB	87	265	ns		[21]
	rs1834481		Japan	TB	87	265	ns		[21]
	rs4937113		Japan	TB	87	265	ns		[21]
	rs2043055		Japan	TB	87	265	ns		[21]
	rs360712		Japan	TB	87	265	ns		[21]
	rs795468		Japan	TB	87	265	ns		[21]

[a] (p_c), corrected p value

[b] OR, odds ratio

[c] pTB, pulmonary TB

[d] H, haplotype

[e] TB, tuberculosis

[f] TST, tuberculin skin test

[g] ns, not significant

[h] HIV+, human immunodeficiency virus positive

References

1. Vidyarani M, Selvaraj P, Prabhu AS, Jawahar MS, Adhilakshmi AR, Narayanan PR: **Interferon gamma (IFNgamma) & interleukin-4 (IL-4) gene variants & cytokine levels in pulmonary tuberculosis.** *Indian J Med Res* 2006, **124:**403-410.

2. Bellamy R: **Genetic susceptibility and resistance to tuberculosis. PhD thesis.** *PhD Thesis.* University of Oxford; 1998.

3. Naslednikova IO, Urazova OI, Voronkova OV, Strelis AK, Novitsky VV, Nikulina EL, Hasanova RR, Kononova TE, Serebryakova VA, Vasileva OA, Suhalentseva NA, Churina EG, Kolosova AE, Fedorovich TV: **Allelic polymorphism of cytokine genes during pulmonary tuberculosis.** *Bull Exp Biol Med* 2009, **148:**175-180.

4. Oral HB, Budak F, Uzaslan EK, Basturk B, Bekar A, Akalin H, Ege E, Ener B, Goral G: **Interleukin-10 (IL-10) gene polymorphism as a potential host susceptibility factor in tuberculosis.** *Cytokine* 2006, **35:**143-147.

5. Ates O, Musellim B, Ongen G, Topal-Sarikaya A: **Interleukin-10 and tumor necrosis factor-alpha gene polymorphisms in tuberculosis.** *J Clin Immunol* 2008, **28:**232-236.

6. Shin HD, Park BL, Kim YH, Cheong HS, Lee IH, Park SK: **Common interleukin 10 polymorphism associated with decreased risk of tuberculosis.** *Exp Mol Med* 2005, **37:**128-32.

7. Thye T, Browne EN, Chinbuah MA, Gyapong J, Osei I, Owusu-Dabo E, Brattig NW, Niemann S, Rusch-Gerdes S, Horstmann RD, Meyer CG: ***IL10*** **haplotype associated with tuberculin skin test response but not with pulmonary TB.** *PLoS ONE* 2009, **4:**e5420.

8. Stein CM, Zalwango S, Chiunda AB, Millard C, Leontiev DV, Horvath AL, Cartier KC, Chervenak K, Boom WH, Elston RC, Mugerwa RD, Whalen CC, Iyengar SK: **Linkage and association analysis of candidate genes for TB and TNFalpha cytokine expression: evidence for association with IFNGR1, IL-10, and TNF receptor 1 genes.** *Hum Genet* 2007, **121:**663-673.

9. Bellamy R, Ruwende C, Corrah T, McAdam KP, Whittle HC, Hill AV: **Assessment of the interleukin 1 gene cluster and other candidate gene polymorphisms in host susceptibility to tuberculosis.** *Tuber Lung Dis* 1998, **79:**83-89.

10. Fitness J, Floyd S, Warndorff DK, Sichali L, Malema S, Crampin AC, Fine PE, Hill AV: **Large-scale candidate gene study of tuberculosis susceptibility in the Karonga district of northern Malawi.** *Am J Trop Med Hyg* 2004, **71:**341-349.

11. Henao MI, Montes C, Paris SC, Garcia LF: **Cytokine gene polymorphisms in Colombian patients with different clinical presentations of tuberculosis.** *Tuberculosis (Edinb)* 2006, **86:**11-19.

12. Tso HW, Ip WK, Chong WP, Tam CM, Chiang AK, Lau YL: **Association of interferon gamma and interleukin 10 genes with tuberculosis in Hong Kong Chinese.** *Genes Immun* 2005, **6:**358-363.

13. Delgado JC, Baena A, Thim S, Goldfeld AE: **Ethnic-specific genetic associations with pulmonary tuberculosis.** *J Infect Dis* 2002, **186:**1463-1468.

14. Lopez-Maderuelo D, Arnalich F, Serantes R, Gonzalez A, Codoceo R, Madero R, Vazquez JJ, Montiel C: **Interferon-gamma and interleukin-10 gene polymorphisms in pulmonary tuberculosis.** *Am J Respir Crit Care Med* 2003, **167:**970-975.

15. Scola L, Crivello A, Marino V, Gioia V, Serauto A, Candore G, Colonna-Romano G, Caruso C, Lio D: **IL-10 and TNF-alpha polymorphisms in a sample of Sicilian patients affected by tuberculosis: implication for ageing and life span expectancy.** *Mech Ageing Dev* 2003, **124:**569-572.

16. Oh JH, Yang CS, Noh YK, Kweon YM, Jung SS, Son JW, Kong SJ, Yoon JU, Lee JS, Kim HJ, Park JK, Jo EK, Song CH: **Polymorphisms of interleukin-10 and tumour necrosis factor-alpha genes are associated with newly diagnosed and recurrent pulmonary tuberculosis.** *Respirology* 2007, **12:**594-598.

17. Prabhu AS, Selvaraj P, Jawahar MS, Adhilakshmi AR, Narayanan PR: **Interleukin-12B & interleukin-10 gene polymorphisms in pulmonary tuberculosis.** *Indian J Med Res* 2007, **126:**135-138.

18. Pacheco AG, Cardoso CC, Moraes MO: ***IFNG* +874T/A, *IL10* -1082G/A and *TNF* -308G/A polymorphisms in association with tuberculosis susceptibility: a meta-analysis study.** *Hum Genet* 2008, **123:**477-484.

19. Tso HW, Lau YL, Tam CM, Wong HS, Chiang AK: **Associations between *IL12B* Polymorphisms and Tuberculosis in the Hong Kong Chinese Population.** *J Infect Dis* 2004, **190:**913-919.

20. Morahan G, Kaur G, Singh M, Rapthap CC, Kumar N, Katoch K, Mehra NK, Huang D: **Association of variants in the *IL12B* gene with leprosy and tuberculosis.** *Tissue Antigens* 2007, **69 Suppl 1:**234-236.

21. Kusuhara K, Yamamoto K, Okada K, Mizuno Y, Hara T: **Association of *IL12RB1* polymorphisms with susceptibility to and severity of tuberculosis in Japanese: a gene-based association analysis of 21 candidate genes.** *Int J Immunogenet* 2007, **34:**35-44.

22. Puzyrev VP, Freidin MB, Rudko AA, Strelis AK, Kolokolova OV: **[Polymorphisms of the candidate genes for genetic susceptibility to tuberculosis in the Slavic population of Siberia: a pilot study].** *Mol Biol (Mosk)* 2002, **36:**788-791.

23. Ma X, Reich RA, Gonzalez O, Pan X, Fothergill AK, Starke JR, Teeter LD, Musser JM, Graviss EA: **No evidence for association between the polymorphism in the 3 ' untranslated region of interleukin-12B and human susceptibility to tuberculosis.** *Journal of Infectious Diseases* 2003, **188:**1116-1118.

24. Akahoshi M, Ishihara M, Remus N, Uno K, Miyake K, Hirota T, Nakashima K, Matsuda A, Kanda M, Enomoto T, Ohno S, Nakashima H, Casanova JL, Hopkin JM, Tamari M, Mao XQ, Shirakawa T: **Association between *IFNA* genotype and the risk of sarcoidosis.** *Hum Genet* 2004, **114:**503-509.

25. Freidin MB, Rudko AA, Kolokolova OV, Strelis AK, Puzyrev VP: **Association between the 1188 A/C polymorphism in the human IL12B gene and Th1-mediated infectious diseases.** *Int J Immunogenet* 2006, **33:**231-232.

26. Akahoshi M, Nakashima H, Miyake K, Inoue Y, Shimizu S, Tanaka Y, Okada K, Otsuka T, Harada M: **Influence of interleukin-12 receptor beta1 polymorphisms on tuberculosis.** *Hum Genet* 2003, **112:**237-243.

27. Sahiratmadja E, Baak-Pablo R, de Visser AW, Alisjahbana B, Adnan I, van Crevel R, Marzuki S, van Dissel JT, Ottenhoff TH, Van D, V: **Association of polymorphisms in IL-12/IFN-gamma pathway genes with susceptibility to pulmonary tuberculosis in Indonesia.** *Tuberculosis (Edinb)* 2007, **87:**303-311.

28. Lee HW, Lee HS, Kim DK, Ko DS, Han SK, Shim YS, Yim JJ: **Lack of an association between interleukin-12 receptor beta1 polymorphisms and tuberculosis in Koreans.** *Respiration* 2005, **72:**365-368.

29. Remus N, El Baghdadi J, Fieschi C, Feinberg J, Quintin T, Chentoufi M, Schurr E, Benslimane A, Casanova JL, Abel L: **Association of *IL12RB1* polymorphisms with pulmonary tuberculosis in adults in Morocco.** *J Infect Dis* 2004, **190:**580-587.

30. Harishankar M, Selvaraj P, Rajeswari DN, Anand SP, Narayanan PR: **Promoter polymorphism of IL-18 gene in pulmonary tuberculosis in South Indian population.** *Int J Immunogenet* 2007, **34:**317-320.
