# Supplementary material for: Analysis of eight genes modulating interferon gamma and human genetic susceptibility to tuberculosis: a case-control association study
Source: BMC Infect Dis. 2010 Jun 7;10:154. doi: 10.1186/1471-2334-10-154 (PMC2891757; doi:10.1186/1471-2334-10-154)
Supplement: Additional file 2 — Candidate genes and polymorphisms analysed in this study. A table containing the results of the single-point association analyses done in this study. [file 1471-2334-10-154-S2.PDF]

Additional file 2 - Candidate genes and polymorphisms analysed in this study.

Gene	Polymorphism		Genotyping	Alleles		Genotype frequencies						Cases vs controls	Power calculations[a]	
						Cases			Controls			p value [c]	MAF [d]	Odds ratio theoretically detectable [e]
				1 [b]	2	11	12	22	11	12	22			
IL4	rs2243248	SNP	SNPlex	G	T	0.13	0.47	0.40	0.11	0.41	0.48	0.08		
	rs2243250 [f]	SNP	TaqMan	C	T	0.28	0.49	0.23	0.24	0.53	0.23	0.37	0.49	1.46
	rs2070874	SNP	SNPlex	C	T	0.35	0.45	0.20	0.36	0.50	0.14	0.05		
	rs2243251	SNP	SNPlex	A	G	0.54	0.38	0.08	0.61	0.31	0.08	0.08		
	rs2243291	SNP	SNPlex	C	G	0.32	0.47	0.21	0.28	0.51	0.20	0.37		
IL10	rs1800890	SNP	SNPlex	T	A	0.05	0.38	0.58	0.06	0.34	0.60	0.34		
	rs1800893	SNP	SNPlex	A	G	0.16	0.53	0.31	0.16	0.46	0.37	0.12		
	rs1800896	SNP	SNPlex	G	A	0.09	0.46	0.45	0.11	0.42	0.47	0.43	0.32	1.49
	rs1800871	SNP	SNPlex	T	C	0.09	0.43	0.48	0.11	0.48	0.42	0.15	0.34	1.48
	rs1800872	SNP	SNPlex	A	C	0.09	0.43	0.48	0.11	0.48	0.42	0.16	0.34	1.48
	rs3024490	SNP	SNPlex	T	G	0.11	0.41	0.48	0.13	0.46	0.42	0.14		
	rs3790622	SNP	SNPlex	T	C	0	0	1	0	0.01	0.99	0.17		

Gene	Polymorphism		Genotyping	Alleles		Genotype frequencies						Cases vs controls	Power calculations[a]	
						Cases			Controls			p value [c]	MAF [d]	Odds ratio theoretically detectable [e]
				1 [b]	2	11	12	22	11	12	22			
	rs3024496	SNP	SNPlex	C	T	0.23	0.53	0.24	0.24	0.48	0.29	0.21	0.48	1.47
	rs3024498	SNP	SNPlex	G	A	0.01	0.24	0.75	0.01	0.19	0.80	0.17		
IL12B	rs730691	SNP	SNPlex	T	C	0.24	0.49	0.27	0.26	0.50	0.24	0.51		
	rs3212217	SNP	SNPlex	C	G	0.09	0.36	0.55	0.06	0.38	0.56	0.18		
	rs3212220	SNP	SNPlex	T	G	0.10	0.40	0.50	0.06	0.44	0.49	0.11		
	D5S2941	STR	CE [g]	L[h]	S[i]	0.09	0.41	0.50	0.08	0.45	0.47	0.27[c], 0.17[j]	0.30	1.49
	rs3213096	SNP	SNPlex	A	G	0	0.003	0.997	0	0.004	0.996	0.82		
	rs2288831	SNP	SNPlex	C	T	0.10	0.40	0.50	0.06	0.45	0.49	0.12		
	rs2853696	SNP	SNPlex	A	G	0.003	0.114	0.883	0.01	0.16	0.83	0.04		
	rs3212227	SNP	SNPlex	C	A	0.09	0.40	0.51	0.06	0.44	0.50	0.23	0.28	1.50
IL12RB1	rs393548	SNP	SNPlex	C	G	0.04	0.32	0.64	0.03	0.32	0.65	0.52	0.19	1.58
	rs2305743	SNP	SNPlex	A	G	0.03	0.33	0.65	0.04	0.29	0.66	0.32		

**Gene**	**Polymorphism**		**Genotyping**	**Alleles**		**Genotype frequencies**						**Cases vs controls**	**Power calculations**[a]	
						**Cases**			**Controls**			**p value** [c]	**MAF** [d]	**Odds ratio theoretically detectable** [e]
				**1** [b]	**2**	**11**	**12**	**22**	**11**	**12**	**22**			
	rs11086087	SNP	SNPlex	C	G	0.02	0.19	0.78	0.01	0.22	0.77	0.5		
	rs429774	SNP	SNPlex	C	T	0.12	0.44	0.44	0.12	0.44	0.44	0.95		
	rs375947	SNP	SNPlex	G	A	0.15	0.44	0.42	0.16	0.43	0.42	0.89	0.37	1.48
IL12RB2	rs11576006	SNP	SNPlex	C	T	0.12	0.50	0.38	0.11	0.44	0.45	0.07		
	rs3762317	SNP	ARMS-PCR	G	A	0.18	0.57	0.24	0.17	0.51	0.31	0.08		
IL18	rs1946519	SNP	SNPlex	A	C	0.11	.50	0.39	0.14	0.48	0.38	0.46		
	rs1946518	SNP	SNPlex	T	G	0.11	0.49	0.39	0.14	0.48	0.38	0.43		
	rs187238	SNP	SNPlex	C	G	0.02	0.23	0.75	0.01	0.24	0.75	0.71		
	rs5744229	SNP	SNPlex	A	G	0.01	0.06	0.93	0.01	0.07	0.93	0.85		
	rs189667	SNP	SNPlex	G	A	0.02	0.23	0.75	0.01	0.24	0.75	0.48		
	rs549908	SNP	SNPlex	G	T	0.02	0.22	0.76	0.01	0.23	0.76	0.88		
WNT5A	rs3796232	SNP	SNPlex	G	C	0.27	0.50	0.23	0.22	0.48	0.30	0.06		

Gene	Polymorphism		Genotyping	Alleles		Genotype frequencies						Cases vs controls	Power calculations[a]	
						Cases			Controls			p value [c]	MAF [d]	Odds ratio theoretically detectable [e]
				1 [b]	2	11	12	22	11	12	22			
	rs1795651	SNP	SNPlex	A	G	0.10	0.41	0.49	0.11	0.43	0.46	0.76		
	rs7624718	SNP	SNPlex	G	A	0.02	0.15	0.83	0	0.18	0.82	0.07		
	rs557077	SNP	TaqMan	C	T	0.22	0.46	0.32	0.21	0.48	0.31	0.85		
	rs566926	SNP	SNPlex	A	C	0.04	0.21	0.75	0.04	0.29	0.67	0.03		
	rs648872	SNP	SNPlex	T	C	0.02	0.27	0.71	0.03	0.27	0.70	0.85		
	rs815541	SNP	SNPlex	G	C	0	0.12	0.88	0.01	0.15	0.84	0.28		
	rs9311564	SNP	SNPlex	G	A	0.01	0.08	0.91	0	0.07	0.93	0.7		
	rs472631	SNP	SNPlex	C	T	0.14	0.42	0.44	0.16	0.46	0.38	0.22		
	rs556874	SNP	SNPlex	A	G	0.09	0.39	0.52	0.10	0.46	0.44	0.06		
	rs11918967	SNP	SNPlex	G	C	0.19	0.46	0.35	0.23	0.48	0.29	0.09		
	rs7622120	SNP	SNPlex	A	G	0.10	0.38	0.52	0.09	0.47	0.44	0.02		
	rs590386	SNP	SNPlex	A	G	0.03	0.22	0.75	0.02	0.21	0.77	0.45		
FZD5	rs10188753	SNP	SNPlex	G	A	0.10	0.40	0.50	0.09	0.41	0.50	0.91		

Gene	Polymorphism		Genotyping	Alleles		Genotype frequencies						Cases vs controls	Power calculations[a]	
						Cases			Controls			p value [c]	MAF [d]	Odds ratio theoretically detectable [e]
				1 [b]	2	11	12	22	11	12	22			
	rs718290	SNP	SNPlex	C	T	0.01	0.18	0.81	0.01	0.15	0.84	0.41		
	rs7582078	SNP	SNPlex	A	C	0.19	0.46	0.35	0.17	0.47	0.36	0.54		
	rs2010400	SNP	SNPlex	T	G	0	0.01	0.99	0	0.01	0.99	0.85		
	rs6708488	SNP	SNPlex	G	A	0.06	0.28	0.66	0.03	0.32	0.65	0.11		
	rs3731568	SNP	SNPlex	C	A	0.05	0.33	0.62	0.05	0.34	0.61	0.97		

[a] With the number of samples available, we had 80% power and 95% confidence given the observed minor allele frequencies to detect the listed effect sizes in the SAC population.

[b] Allele 1 is the minor allele

[c] From a genotype-based, two-tailed χ^2 or Fisher's exact test.

[d] MAF, minor allele frequency

[e] Effect sizes greater than this value for the SNP listed can be excluded in the SAC population based on the negative results of this study. Therefore, this study had enough power to validate previously reported effect sizes.

[f] Underlined rs-numbers indicate polymorphisms previously associated with TB in single-point or haplotype analyses.

[g] CE, capillary electrophoresis.

[h] L, longer repeats with $(ATT)_9$ and $(ATT)_{10}$.

[i] S, shorter repeats with $(ATT)_7$ and $(ATT)_8$.

[j] p value of comparison of genotypes with and without S allele.
